# The effect of histopathologic and clinical features on allograft survival in renal transplant patients with antibody-mediated rejection

**DOI:** 10.1080/0886022X.2016.1244073

**Published:** 2016-10-24

**Authors:** Tulin Akagun, Halil Yazici, Yasar Caliskan, Yasemin Ozluk, Sevgi Sahin, Aydin Turkmen, Isın Kılıcaslan, Mehmet Sukru Sever

**Affiliations:** a Division of Nephrology, Department of Internal Medicine, Istanbul Faculty of Medicine, Istanbul University, Istanbul, Turkey;; b Department of Pathology, Istanbul Faculty of Medicine, Istanbul University, Istanbul, Turkey;; c Nephrology Clinic, Acibadem Atakent Hospital, Istanbul, Turkey

**Keywords:** Renal transplantation, antibody-mediated rejection, allograft survival, transplant biopsy, serum creatinine

## Abstract

**Background:** Antibody-mediated rejection is a frequent cause of graft failure; however, prognostic indications of this complication have not been well defined. The aim of this study was to evaluate the association of histopathological and clinical features and to determine the effect of these findings on allograft survival in patients with AMR.

**Methods:** Fifty-two patients suffered from AMR (30 male; mean age 39 ± 11 years) were included in the study. Data were investigated retrospectively and graft survival was analyzed. All transplant biopsies were evaluated according to Banff 2009 classification.

**Results:** Of the 52 cases, 45 were transplanted from living-donors. Twenty-one patients were diagnosed in the first 3-months after transplantation. Graft survival was 65% at 12 months and 54% at 36 months. Mean serum creatinine at time of biopsy was 3.8 ± 3.6 mg/dL. Thirty-five of the 52 cases showed diffuse C4d positivity, 12 cases showed focal and 5 remained C4d negative. One of the patients died, 13 experienced graft loss and 38 survived with functioning grafts. Serum creatinine levels at time of biopsy were correlated with graft survival (*p* = .021: OR = 1.10: 95 % CI = 1.015–1.199). In terms of the impact of pathological findings; tubulitis (*p*=.007: OR = 2.62: 95 % CI = 1.301–5.276), intimal arteritis (*p*=.017: OR = 2.85: 95% CI = 1.205–6.744) and interstitial infiltration (*p*=.004: OR = 3.37: 95% CI = 1.465–7.752) were associated with graft survival.

**Conclusions:** Serum creatinine at time of biopsy, tubulitis, intimal arteritis and interstitial infiltration were significantly associated with graft survival. Antibody-mediated rejection is associated with reduced long-term graft survival.

## Introduction

Antibody-mediated rejection is a frequent cause of graft failure; however, prognostic indications of this complication have not been well defined. Although it is typically a response to donor HLA antigens expressed on endothelial cells, antibody mediated rejection (AMR) can also occur to non-HLA antigens.^1^^,^^2^

Previous studies have reported an incidence of AMR between 5.6% and 23%.^3–6^ AMR is relatively rare after kidney transplantation.^7^^,^^8^ Nonetheless, the incidence among high-risk, sensitized patients still exceeds 25%.^9^^,^^10^^,^^11^ The incidence of AMR in patients with renal transplant varies depending on the transplantation treatment protocol applied to high-risk, sensitized patients and the method used to identify the donor-specific antibody (DSA), but has been reported at 0–8.9%.^12^ Major histocompatibility complex (MHC) class 1 and 2 antigens are the main target of alloantibodies in transplantation. Minor histocompatibility antigens and also various non-HLA antigens can trigger development of antibodies.^13^

Diagnosis of AMR is based on three basic criteria: (1) evidence of allograft dysfunction, (2) antibody-dependent activation of classic complement system and morphological evidence of acute tissue injury (peritubular capillary C4d deposition) and (3) determination of circulating DSAs.^14^ According to Banff criteria, diagnosis of acute AMR is made on the basis of C4d positivity in the presence of circulating anti-donor antibodies and findings of tissue injury (acute tubular necrosis-like minimal inflammation (ptc/g >0) and/or thrombosis and arteritis).^15^ Treatment of AMR after diagnosis is based on 4 essential mechanisms; (1) suppression of T cell response, (2) removal of circulating antibodies, (3) inhibition of antibodies and (4) suppression of B cells.^14^^,^^16^

Peritubular capillary C4d deposition observed in rejections in the early post-transplant period is associated with AMR and has been found to be correlated with poor graft survival.^17^ Comparison of patients with no history of rejection or with a history of acute cellular rejection has revealed a significant decrease in long-term survival of grafts undergoing AMR.^14^^,^^18^

The aim of this study was to evaluate the association of histopathological and clinical features and to determine the effect of these findings on allograft survival in the patients with AMR.

## Subjects and methods

### Patients

Among the patients transplantations were performed from January 2006 to May 2012, 52 patients were diagnosed to be complicated by AMR. These patients were retrospectively evaluated on the basis of clinical records and enrolled in this study.

Information concerning demographic (age, gender, etc.), clinical (the etiology of chronic kidney disease (CKD), duration of hemodialysis (HD), the type of renal replacement therapy, therapeutic features), laboratory (serum creatinine (Cr), hemoglobin, C-reactive protein (CRP), proteinuria, immunological characteristics) and histopathological fetures were retrospectively analyzed by reviewing the patient files. All transplant biopsies were evaluated by experienced nephropathologist according to Banff 2009 classification.

### Statistical analysis

Numerical variables were expressed as mean values and standard deviations (mean ± SD) or median values. Graft survival was evaluated using the Kaplan–Meier method, and the Cox model was used for multivariate analysis. Repeated measures were evaluated using Pillai’s trace test and the Bonferroni test was used for comparison of two groups. A *p* value of < .05 was considered statistically significant. Statistical analysis was performed by Statistical Package for Social Sciences for Windows version 15.0 (SPSS Inc., Chicago, IL) software.

## Results

### Demographic and clinical features

Fifty-two renal transplant patients with AMR (30 male; mean age 39 ± 11 (range 20–58) years) were included in the study. The patients’ demographic characteristics are shown in Table 1. Mean age at time of diagnosis was 39.3. The majority of donors were living-donors (86.5%).

**Table 1.** Demographic and clinical features of the renal transplant patients with AMR.

Parameter	Patients (*n* = 52)
Age (mean ± SD)	39.3 ± 11.2
Male/Female	30/22
Primary disease	
Unknown	13 (25%)
Glomerulonephritis	10 (19.2%)
Pyelonephritis	7 (13.5%)
Vesicoureteral reflux	6 (11.5%)
Hypertension	5 (9.6%)
Diabetes mellitus	4 (7.7%)
Congenital urologic anomalies	4 (7.7%)
Policystic kidney disease	2 (3.8%)
Bilateral cortical necrosis	1 (1.9%)
Time on dialysis (months)	46,5 ± 57,5
Renal replacement therapy	
Hemodialysis (HD)	35 (67.3%)
Peritoneal dialysis (PD)	3 (5.7%)
HD + PD	2 (3.8%)
Pre-emptive	5 (9.6%)
Unknown	7 (13.5%)
Donor type	
Living donor	45 (86.5%)
Deceased donor	7 (13.5%)

Six patients (11.5%) had a history of previous renal transplantation and one of them received both a liver and a kidney transplant. Ten female patients' past medical history were characterized by pregnancies, while 21 patients received blood transfusion.

### Laboratory data

Pre-transplant immunological characteristics of the patients are shown in Table 2. The median number of HLA mismatches was three. Twelve (%30) of the 40 patients had received no induction therapy. Fifteen of the patients had received anti-tymocit globulin (ATG), 13 received basiliximab.

**Table 2.** Pre-transplant immunological characteristics of the renal transplant patients with AMR.

Parameter	Number of patients
ABO (compatible/incompatible)	41/0
CDC CM (negative/positive)	28/0
T cell FC CM (negative/positive)	32/0
B cell FC CM (negative/positive)	29/3
PRA class 1 (negative/positive)	33/4
PRA class 2 (negative/positive)	35/2
HLA mismatches (mean/minimum-maximum)	3.86 (2–6)

CM: cross-match; FC: flow cytometry; PRA: panel reactive antibody.

Patients’ immunological characteristics and laboratory data are shown in Table 3.

**Table 3.** Laboratory data and immunologic characteristics of the patients at the time of biopsy.

	*n*	Mean ± SD	Range
Serum Cr (mg/dL)	52	3.8 ± 3.6	1–21
Hb (g/dL)	48	9.7 ± 1.8	6.3–14.1
CRP (mg/dL)	25	14.2 ± 27.9	0.3–138
Proteinuri (g/gün)	26	2.98 ± 4.16	0.2–20
PRA class 1 (%)	35	11.2 ± 15.6	5–75
PRA class 2 (%)	35	22.4 ± 27.4	3–87

Cr: creatinine; CRP: C-reactive protein; Hb: hemoglobin; PRA: panel reactive antibody.

AMR was diagnosed at a mean of 34.5 (range 0–192) months post-transplantation Acute AMR was diagnosed in 21 cases (40.4%). Mean serum creatinine level was 3.8 ± 3.6 (1–21) mg/dL. Proteinuria was 2.98 ± 4.16 g/day at time of biopsy (Table 3). Thirty-five of the 52 patients were available for PRA analysis in the peribiopsy period (Table 3). Positivity for PRA class 1 and 2 antibodies at the time of biopsy was determined in 18 (51.4%) of the 35 patients for whom data were available. Of the 26 subjects with anti-HLA antibodies, 6 cases donor-specific anti-HLA antibodies (DSA) were detected in 6 cases (Table 4).

**Table 4.** PRA and DSA results at time of biopsy.

	*n*	%
PRA class 1		
Positive	7	20
Negative	28	80
Total	35	100
PRA class 2		
Positive	15	42.9
Negative	20	57.1
Total	35	100
DSA		
Positive	6	23
Negative	20	77
Total	26	100

PRA: panel reactive antibody; DSA: donor specific antibody.

### Histopathologic data

The histopathology of the allograft biopsy specimens with AMR is shown in Table 5. Of the 43 AMR biopsy specimens, 9 were v1 (intimal arteritis), 3 were v2 and 3 were v3. Interstitial inflammation (i1-i3) was present in 45 biopsy specimens, tubulitis (t1-t3) in 40, glomerulitis (g1-g3) in 34 and peritubular capillaritis (ptc1-ptc3) in 41. C4d deposition in PTC was observed in 47/52 cases as either diffuse (*n* = 35) or focal (*n* = 12) staining (Table 5).

**Table 5.** The histopathology of 52 allograft biopsies with AMR.

Case	V	İ	t	G	ptc	C4d
Banff classification score						
1	0	1	1	1	1	Diffuse
2	0	2	2	2	3	Diffuse
3	0	1	1	0	2	Diffuse
4	0	2	1	1	3	Diffuse
5		1	1	1	2	Diffuse
6	0	1	2	0	1	Negative
7	0	1	1	2	0	Negative
8	0	1	1	1	1	Diffuse
9		2	2	0	2	Diffuse
10	0	1	1	1	1	Negative
11	0	2	1	2	3	Diffuse
12	0	1	2	1	2	Diffuse
13	0	2	1	2	2	Focal
14		1	1	0	1	Diffuse
15		2	1	3	2	Diffuse
16	0	2	1	1	2	Diffuse
17	0	2	1	3	2	Focal
18						Focal
19	0	2	2	0	1	Diffuse
20		1	1	1	2	Diffuse
21		1	1	0	0	Diffuse
22	1	3	3	1	1	Negative
23	1	2	3	0	1	Diffuse
24	0	1	1	1	3	Focal
25	0	2	1	2	1	Negative
26		0	0			Diffuse
27	3	2	0	1	0	Diffuse
28	1	3	1	2	2	Diffuse
29	2	3	3	0	3	Focal
30	0	1	0	1	0	Focal
31	1	3	3	0	2	Diffuse
32	2	2	1	2	1	Diffuse
33	1	1	0	2	2	Focal
34	1	1	0	1	1	Diffuse
35	3	0	0	1	0	Diffuse
36	3	3	3	1	2	Diffuse
37	2	2	1	1	1	Focal
38	0	0	0	2	1	Diffuse
39	1	1	1	0	2	Diffuse
40	1	3	3	0	3	Diffuse
41	0	3	2	1	2	Diffuse
42	0	2	2	1	1	Focal
43	0	0	0	1	1	Focal
44	0	3	2	2	2	Diffuse
45	0	1	1	0	2	Focal
46	0	3	2	0	2	Diffuse
47	0	1	0	0	0	Diffuse
48	0	0	0	2	0	Diffuse
49	3	3	3	3	3	Diffuse
50	0	3	2	0	1	Diffuse
51	0	0	0	0	0	Diffuse
52	1	1	1	2	0	Focal

V: intimal arteritis; İ: interstitial infiltration; t: tubulitis; G: glomerulitis; ptc: peritubular capillaritis; C4d: C4d deposition in PTC.

### Therapeutic and clinical features

Twenty-eight of the 52 patients were receiving tacrolimus (FK)+mycofenalic acid derivative (M)+prednisolone (P), 17 patients receiving cyclosporine (C) +M + P, 4 patients receiving C + azathiopurine + P, 1 patient receiving sirolimus + M + P, 1 patient receiving FK + M and 1 receiving FK + P as basal immunosuppression. The patients were determined to have received one or more of pulse steroid (CS), anti-thymocyte globulin (ATG), intravenous immunoglobulin (IVIg), rituximab (R), plasmapheresis (PP), eculizimab (E) and alemtuzumab (A) therapies. Two of 52 patients received pulse steroid, 2 of the patients received CS + ATG, 11 patient received CS + PP, 4 patient CS + R + PP, 7 patient CS + ATG + PP, 2 patient CS + ATG++IVIg, 8 patient CS + IVIg + PP, 3 patient CS + ATG + R + PP, 5 patient CS + IVIg + R + PP, 5 patient CS + ATG + IVIg + PP, 1 patient CS + IVIG + R + PP + E + A, 1 patient CS + ATG + IVIg + R + PP + E and 1 patient received CS + ATG + IVIg + R + PP (Table 6). Thirty percent of our cases did not receive induction therapy. The KDIGO guidelines recommend therapy with IL-2 receptor agonist to low risk and ATG to high-risk patients.^19^ The fact that induction therapy was not administered in 30% of our cases may have played a role in the development of AMR.

**Table 6.** Anti-rejection therapies received by AMR patients.

Anti-rejection therapy	*n* (52)
CS + ATG + IVIg + R+PP + E	1
CS + IVIg + R+PP + E+A	1
CS + ATG + IVIg + R+PP	1
CS	2
CS + ATG	2
CS + ATG + IVIg	2
CS + ATG + R+PP	3
CS + R+PP	4
CS + IVIg + R+PP	5
CS + ATG + IVIg + PP	5
CS + ATG + PP	7
CS + IVIg + PP	8
CS + PP	11

A: alemtuzumab; ATG: anti-thymocyte globulin; E: eculizimab; IVIg: intravenoz immunoglobulin; CS: corticosteroid pulse; PP: plasmapheresis; R: rituximab.

Serum creatinine levels were investigated at the 1st and 2nd weeks and 1st and 3rd months after the treatment. Serum creatinine levels were 3.1 ± 1.9 mg/dL in the 1st week, 2.8 ± 2.2 mg/dL in the 2nd week, 2.4 ± 2.1 mg/dL at the 1st month and 2.2 ± 1.4 mg/dL at the 3rd month. When post-treatment responses were compared using Pillai’s trace test, serum creatinine levels declined after treatment, although the decrease did not achieve statistical significance (*p* = .063).

Thirty-eight of the 52 patients recovered renal allograft functions after anti-rejection therapy. One of the patients (1.9%) died while 13 (25%) experienced graft loss.

Mean final serum creatinine level in the patients monitored was 1.8 ± 0.9 (1–5) mg/dL. When serum creatinine levels were divided on the basis of <2 mg/dL and ≥2 mg/dL, 24 patients (63.2%) had final control serum creatinine levels ≥2 mg/dL.

Factors that might affect graft survival were examined using Cox regression analysis. When the two groups were compared using log rank analysis in terms of time to rejection (acute or chronic cases) a statistically significant difference was determined in terms of renal survival (*p* = .000).

Patients with positive PRA (*n* = 18) had a worse graft survival as compared to those with negative PRA (*n* = 17) (*p* = .040).

Serum creatinine levels investigated during biopsy emerged as a factor affecting graft survival (*p* = .021; OR = 1.10; 95% confidence interval lower-upper thresholds =1.015–1.199).

Examination of biopsy findings in graft survival identified tubulitis, intimal arteritis and interstitial inflammation as effective factors. The relevant findings are given in Table 7.

**Table 7.** Pathological characteristics affecting graft survival.

	*p*	OR	At %95 confidence
Tubulitis	.007	2.62	1.301–5.276
Intimal arteritis	.017	2.85	1.205–6.744
Interstitial infiltration	.004	3.37	1.465–7.752

No significant difference was determined in terms of graft survival when we divided our patients into two groups on the basis of glomerulitis + peritubular capillaritis (total g + ptc) score,^20^ a novel form of assessment in patients with renal transplant, (patients with g + ptc score ≤3 and >3).

Three groups were compared in terms of C4d staining (diffuse, focal staining and no staining) using Log rank analysis. C4d staining was shown to have no effect on graft survival (*p* = .287).

**Figure 1.**
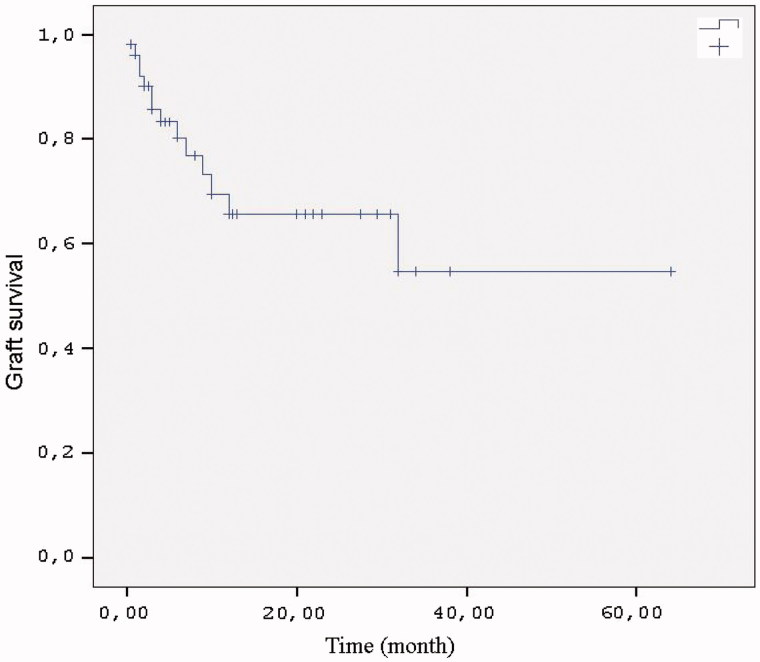
Graft survival (up to latest assessment after rejection).

Kaplan–Meier survival analysis revealed graft survival rates of 65% and 54% at the 12th and 36th months, respectively (Figure 1).

## Discussion

Acute and chronic antibody-mediated rejections are stubborn entities with significant effects on patient and graft survival. Compared with the patients with no history of rejection or cases who have experienced acute cellular rejection, there is a significant decrease in long-term graft survival who have undergone AMR.^14^

One patient (1.9%) died in this study. Graft loss occurred in 26.9% of patients despite treatment. Twelve and 36 month graft survival rate was 65% and 54%, respectively. Retrospective studies in the literature have reported graft survival rates at 1 year of 70–100% in patients receiving plasmapheresis (PP)-based combination therapies.^21^ Different treatment protocols were applied in these studies and our own research, and our graft survival rate was lower than those reported elsewhere. This may be associated with our higher case numbers. In a study of 16 patients, Rocha et al. reported a graft survival rate at 569 days of 81% following administration of PP + IVIg.^4^ Faguer et al. reported a graft survival rate at 10 months of 75% following plasmapheresis + rituximab therapy in a study of 8 patients.^22^ In a study published in 2007, estimated patient survival was 99% and graft survival 80% over a mean 18-month follow-up period.^23^ The patient numbers in these studies concerning graft survival were lower than those in our study. In addition, since timing of the diagnosis in our study was 34.5 months, our patients were diagnosed later; which resulted in a more severe histopathological finding. In clinical practice, when graft dysfunction occurs such as to warn the clinician, the rejection process is generally advanced and serious histopathological injury has already occurred. Since antibody screening or protocol biopsy was not performed in all our patients diagnosis was relatively late; this may have affected the treatment success. In addition, 11.5% of our patients underwent second transplantation and were therefore at immunological risk.

Mean serum creatinine values examined at a mean of 13.6 months after treatment in patients were 1.8 ± 0.9 mg/dL. Previous studies with lower patient numbers have reported mean serum creatinine levels of 1.6–1.7 mg/dL.^4^^,^^22^^,^^24^

Mean time to diagnosis in our cases was 34.5 months, therefore can be considered as late acute rejection. One study comparing C4d positive and negative cases with late acute rejection reported mean diagnosis times of 38.5 and 45.8 months, respectively.^17^ A significant difference was determined in terms of graft survival between cases in which rejection occurred in the acute period and those in which it occurred in the chronic period (*p* = .000), with longer graft survival in cases diagnosed within the first 3 months post-transplant. This may be attributed to graft prognosis being poorer in episodes of late rejection.^4^ In addition, although no effect of interstitial fibrosis and tubular atrophy on graft survival was revealed in this study, long-term exposure to calcineurin inhibitors in rejections occurring in the later period after transplantation may have caused chronic injury in the graft. This may contribute to the low graft survival levels.

PRA class 1 or class 2 antibody positivity was determined in 51.4% of our patients, and DSA positivity was noted in 23%. One study of 17 patients diagnosed with AMR reported PRA positivity in 59% of the cases and DSA positivity in 25%.^25^ Another study determined a DSA positivity level of 25%.^17^ These results are comparable with our findings. DSA positivity, one of the diagnostic criteria for AMR, is not detected in all cases. This may be associated with the presence of non-HLA antibodies that may play a role in the development of rejection.^13^^,^^26^

HLA matching is generally higher in transplants from living donors. Cecka et al. reported approximately 11% of living donor transplants were HLA-matched and 10-year graft survival was 74% compared to 58% for HLA-mismatched transplants.^27^ Mean serum creatinine level at time of biopsy in our patients was 3.8 mg/dL. In one study, patients were classified on the basis of C4d positivity. Mean serum creatinine in the C4d negative group at time of biopsy was 3.7 mg/dL, compared to 4.6 mg/dL in the C4d diffuse positive group.^28^ Different results may be attributed to the heterogeneous nature of the study groups. One of the important results from our research is to find a correlation between mean serum creatinine level at time of biopsy and graft survival. Another study showed that graft function and a high PRA level during biopsy were predictors of graft loss.^28^ Similarly, we have also found that graft survival was significantly shorter in PRA positive cases at time of biopsy.

Studies investigating the association between C4d staining in peritubular capillaries and graft survival have reported varying results. One study reported, in agreement with our own findings, lower graft survival in a C4d-positive group, but concluded that this did not achieve statistical significance.^28^ One study published in 2011 showed that C4d staining was not correlated with graft or patient survival.^29^ Another study reported that C4d staining in peritubular capillaries in cases of late acute rejection had no predictive power in terms of graft survival.^17^ In contrast to these findings, one study published in 2010 determined an association between C4d positivity (diffuse or focal) and poor graft survival.^30^ It is impossible to reach a more definitive conclusion regarding the relation between C4d staining in peritubular capillaries and graft survival on the basis of the available results. Further, more comprehensive studies with more cases and a longer follow-up time are needed. In addition, since C4d accumulation can fluctuate in cases in the early period, it should be remembered that it may not always be possible to establish a sound correlation with AMR.

In terms of the effect of biopsy findings on graft survival, we identified intimal arteritis, tubulitis and interstitial infiltration as influencing factors. One study investigated the relation between Banff ’97 classification and clinical survival and determined that presence of intimal arteritis was a predictive factor increasing the probability of graft loss in acute vascular rejection. The authors reported that among specific pathological lesions, intimal arteritis was the most important predictor of poor outcome.^31^ Another study reported a correlation between intimal arteritis and poor prognosis in AMR.^32^ These results are compatible with our own findings. We also identified tubulitis and interstitial infiltration as factors affecting graft survival. Accompanying acute cellular rejection was identified in 19 (36.5%) of our cases. This finding may be associated with a combination of cellular and humoral rejection being associated with poor graft survival.^33^

When we divided our patients into two groups on the basis of glomerulitis + peritubular capillaritis (total g + ptc) score^20^ (g + ptc score ≤3 and >3), one recent mode of assessment in biopsies of renal transplant patients, we determined no significant difference in terms of graft survival. A recent study published by Sis et al. in 2012 compared patients with a g + ptc score of 4–5 with those with a g + ptc score of 1–3 and 0 and reported significantly lower graft survival in patients with a g + ptc score of 4–5. That study reported that a g + ptc score greater than zero in the presence of DSA can be added to the definition of AMR in Banff classalcification without the need for C4d positivity.^20^ Our results may not have achieving statistical significance due to our low case numbers.

In our research, ATG treatment was identified as a therapeutic option by itself increasing graft survival. Previous studies have used ATG as an adjuvant therapy in the treatment of AMR with characteristics of humoral and cellular rejection.^4^^,^^34–37^ The reason why ATG was identified as an effective factor in graft survival in our study was the presence of AMR cases accompanied by acute cellular rejection.

Examination of all the treatments applied to our patients together revealed that IVIg also has a positive effect on graft survival. IVIg therapy has generally been administered in combination with plasmapheresis and rituximab in previous studies.^4^^,^^22^^,^^24^ Additionally, in Rocha et al.’s study, plasmapheresis and IVIg therapy was administered to 14 of 16 patients diagnosed with AMR, all patients received steroid pulse therapy and 4 patients received anti-lymphocyte therapy (OKT-3 or ATG) due to accompanying acute cellular rejection, and graft survival at 1 year was determined at 81%.^4^ Plasmapheresis was administered to 46 of the 52 patients in our research, and IVIg to 23. Combination plasmapheresis + IVIg therapy was thus administered to some of our patients. Slatinska et al. compared a plasmapheresis + IVIg combination with plasmapheresis alone and reported that combined treatment was more effective than plasmapheresis alone.^38^

In conclusion, AMR has a significant and negative effect on graft survival. Diagnosis once histopathological damage occurs in the graft and when serum creatinine levels rise reduces the probability of successful treatment. In our study, serum creatinine level at time of biopsy, tubulitis, intimal arteritis and interstitial infiltration adversely affected graft survival. Earlier diagnosis is important in order to improve success in treatment of AMR and graft survival. We conclude that periodic post-transplant PRA and DSA monitoring may be beneficial for that purpose.
